# Stage-Specific Value of Carbohydrate Antigen 19-9 and Carcinoembryonic Antigen Serum Levels on Survival and Recurrence in Pancreatic Cancer: A Single Center Study and Meta-Analysis

**DOI:** 10.3390/cancers12102970

**Published:** 2020-10-14

**Authors:** Labrinus van Manen, Jesse V. Groen, Hein Putter, Martin Pichler, Alexander L. Vahrmeijer, Bert A. Bonsing, J. Sven D. Mieog

**Affiliations:** 1 Department of Surgery, Leiden University Medical Center, 2300RC Leiden, The Netherlands; l.van_manen@lumc.nl (L.v.M.); j.v.groen@lumc.nl (J.V.G.); a.l.vahrmeijer@lumc.nl (A.L.V.); b.a.bonsing@lumc.nl (B.A.B.); 2 Department of Medical Statistics, Leiden University Medical Center, 2300RC Leiden, The Netherlands; h.putter@lumc.nl; 3 Division of Clinical Oncology, Medical University of Graz, 8036 Graz, Austria; martin.pichler@medunigraz.at

**Keywords:** pancreatic neoplasms, carcinoembryonic antigen, carbohydrate antigen 19.9, survival, recurrence

## Abstract

**Simple Summary:**

Pancreatic cancer is one of the most aggressive cancers with a poor survival. Only the minority of patients can be treated by extensive surgery, which is associated with high morbidity. Therefore it could be helpful to identify which patients are at risk of early recurrence and associated poor survival in order to optimize treatment strategies for individual patients. Serum tumor markers, which are readily available and easily implicated in the clinical workflow, are such additional tools. In this study, tumor markers carbohydrate antigen 19-9 (CA19-9) and carcinoembryonic antigen (CEA) have been studied and results have been compared with existing literature by performing a systematic literature search, as current literature is lacking a complete overview of the prognostic value of both markers. Elevated CA19-9 serum level appear to be an independent prognostic factor for poor survival and early recurrence in pancreatic adenocarcinoma patients, whereas the prognostic value of CEA is disputable.

**Abstract:**

This study aimed to determine the stage-specific prognostic value of carbohydrate antigen 19-9 (CA19-9) and carcinoembryonic antigen (CEA) serum levels at diagnosis on overall survival (OS) and time to local recurrence or distant metastases in patients with pancreatic ductal adenocarcinoma (PDAC). Consecutive PDAC patients, discussed at multidisciplinary team meetings from 2013 through 2017, were reviewed. Prognostic factors were stage-specific (resection vs. advanced PDAC) evaluated in Cox proportional hazard models. Additionally, a systematic literature search and meta-analysis was performed, as current literature is lacking a complete overview of used cut-off values and the added value of CEA as prognostic marker. In the retrospective cohort, elevated CA19-9 (>305 kU/L) level was independently associated with poor OS (Hazard ratio (HR): 1.72(1.31–2.26)) and early recurrence (HR: 1.74(1.06–2.86)), whereas CEA was not significantly associated. The meta-analysis showed that both elevated CA19-9 and CEA serum levels were predictors for poor OS (pooled HR: 1.29(1.17–1.42) and HR: 1.51(1.33–1.73), respectively). In the resected cohort, elevated CA19-9 level was significantly associated with early recurrence (pooled HR: 2.41(1.77–3.29)), whereas CEA was not. Elevated CA19-9 serum level appear to be an independent prognostic factor for poor OS and early recurrence in PDAC patients, whereas the prognostic value of CEA is disputable.

## 1. Introduction

Pancreatic ductal adenocarcinoma (PDAC) is one of the most aggressive cancers with an overall 5-year survival rate up to 8% [1]. Only the minority of patients can be treated by extensive surgery [2]. As the diagnosis and treatment of PDAC is sometimes difficult to establish, patients are in general discussed during multidisciplinary team (MDT) meetings. Advances in neoadjuvant treatment have shown promising results in preoperative downstaging of locally advanced disease, resulting in improved survival after surgery [3,4]. Therefore, it could be helpful to identify which patients are at risk of early recurrence and associated poor survival after extensive surgery and consequently in which patients the MDT should favor neoadjuvant therapy. Moreover, tumor biology could be heterogenous in patients with similar tumor anatomy, and should be taken into consideration during the clinical work-up [5,6]. Additional tools, which should be readily available and easily implicated in the clinical workflow, to determine the optimal treatment strategy for the individual patient would add significant value to this multidisciplinary decision making process.

Serum tumor markers are such additional tools and their role as biological markers have been studied for several cancer types. Carbohydrate antigen 19-9 (CA19-9) and carcinoembryonic antigen (CEA) are the most studied tumor markers in pancreatic cancer and are both associated with disease stage and overall survival (OS) in patients who underwent pancreatic tumor resection. However, few studies reported on CA19-9 as marker for recurrence [7,8,9], whereas no studies evaluated CEA as a marker for recurrence. Moreover, little is known on the prognostic value of the combination of both tumor markers on OS and time to recurrence in unselected patients discussed at MDT meetings; thus not only in patients who underwent resection. Furthermore, current literature is lacking a complete overview of the prognostic value of both markers and the associated cut-off values that can be incorporated in patient and treatment selection allocation.

The primary aim of this study was to determine the prognostic value of CA19-9 and CEA serum levels on OS and time to recurrence in PDAC patients, discussed in MDT meetings. The secondary aim was to pool existing data on the prognostic value of CA19-9 and CEA serum levels. Therefore, we performed a systematic literature search and meta-analysis, to compare our findings with the existing literature.

## 2. Methods

### 2.1. Patient Selection

All consecutive patients discussed at MDT meetings from January 2013 through December 2017 at Leiden University Medical Center (Leiden, The Netherlands), were reviewed. Only patients diagnosed with PDAC, confirmed by pathological examination, or patients with a strong suspicion of PDAC, in case resection or biopsy could or was not performed, were included. Patients with resectable disease (as decided by the MDT), who did not want to undergo surgery or were deemed unfit for surgery were excluded from the analyses, because resectability, i.e., tumor stage, could not be proven by surgical exploration. Approval of the local Medical Research and Ethical Committee was obtained for this study (protocol number: G17.059) on 4 June 2018.

### 2.2. Definitions

Laboratory findings (CEA, CA19-9 and total bilirubin) were defined as the last measured value before an MDT meeting. Preoperative staging of PDAC was performed according to the American Joint Committee on Cancer (AJCC; 7th edition) [10]. At MDT meetings at least one medical specialist of the following departments was present: Medical Oncology, Radiology, Hepatopancreaticobiliary Surgery, Gastroenterology and Pathology. All patients underwent radiological staging using Computed Tomography (CT) or Magnetic Resonance Imaging (MRI) or both in order to assess the resectability of the tumor. Tumor size was determined as the largest diameter in the transversal direction on CT or MRI.

Patients who did not undergo resection because of locally advanced (LAPC) or presence of metastases (M+), were categorized in the advanced PDAC group. LAPC was defined according to the Dutch Pancreatic Cancer Group (DPCG, 2012), as either tumor abutment of the superior mesenteric artery, celiac axis or common hepatic artery >90° of the circumference of the vessel wall or tumor involvement of the superior mesenteric vein/portal vein vessel wall resulting in occlusion or >270° contact.

R0 resection was defined as a free margin of >1 mm on microscopic level, in accordance with the Royal College of Pathologists guidelines [11]. OS was calculated from the date of the first suspicion of pancreatic cancer on CT or MRI to the date of death (event) or last follow-up (censored). The overall time to recurrence was calculated as the interval between the date of surgery and time of recurrence. Disease recurrence (local or distant) was confirmed by imaging (CT, MRI, or PET-CT), which was performed in case of clinical symptoms suspected for disease recurrence [12]. In case a patient died without evidence of recurrence (censored), the date of last follow-up imaging or last follow-up without clinical signs of recurrent disease was used.

### 2.3. Statistical Analysis

Continuous variables are presented as mean (standard deviation (SD)) in normal distributed data or median (interquartile range (IQR)) in non-normal distributed data. Categorical variables are presented as absolute numbers and percentages. Chi Squared test (for categorical variables), One-Way ANOVA (for normal distributed continuous variables), and Kruskal-Wallis test (for non-normal distributed continuous variables) were used to compare the patients and tumor characteristics between the resected PDAC, intraoperative PDAC and postoperative PDAC groups. Missing tumor marker values were imputed 50 times based on relevant prognostic factors, including sex, age, tumor size, survival, tumor stage, and resectability [13]. The Kaplan-Meier method was used to estimate median OS and time to recurrence. The curves were compared using the log-rank test. 

CA19-9 and CEA were considered elevated if above 27 kU/L or 3 µg/L, respectively, according to the laboratory cut-off values commonly used in our center. Moreover, other clinically relevant cut-off values, CEA>7 µg/L and CA19-9>305 kU/L, as recently detected by our group, were also used in the analyses as well as combined cut-off values [14].

Potential prognostic factors were first evaluated in an univariable Cox proportional hazard model. Variables with P-value below 0.200 were further evaluated in a multivariable Cox proportional hazard model, except for CA19-9 and CEA, which were evaluated irrespective of statistical significance. For the tumor markers CA19-9 and CEA, the most significant cut-off values in the univariate analysis (standard Kaplan-Meier curve) and preoperative bilirubin levels were used in the multivariable analysis. 

A P-value below 0.05 (two-sided) was considered to be statistically significant. Statistical analysis was performed using IBM SPSS Statistics for Windows, version 23.0 (IBM Corp., Armonk, NY, USA). 

### 2.4. Systematic Review of the Literature

A systematic literature search was performed in PubMed for full-text articles reporting on the prognostic value of preoperative CA19-9 and CEA levels for OS and time to recurrence in PDAC patients. The search strategy consisted of the terms CA19-9, CEA, survival, recurrence, pancreatic cancer and/or synonyms. Screening of titles, abstract and subsequently full-text articles for eligibility and the data extraction was performed by two independent reviewers (L.M. and J.V.G). Only studies that report a hazard ratio (HR) in multivariable analysis were included in the pooled analysis. To assess heterogeneity between the studies the I^2^ statistic was used. As the number of included studies was limited and cohort sizes varied, inverse-variance random-effect models were used to calculate pooled effects. Publication bias was assessed by funnel plots. All analyses were performed using Review Manager (RevMan 5; The Cochrane Collaboration, London, UK). The systematic review and meta-analysis was performed according to the PRISMA guidelines [15].

## 3. Results

### 3.1. Patient and Tumor Characteristics

In total, 420 consecutive PDAC patients were discussed at our MDT meetings, of which 45 patients did not want to undergo further medical analysis or were deemed unfit for surgery. Three hundred 75 patients were available for analysis: 151 (40%) patients underwent resection and 224 (60%) patients did not undergo tumor resection because of either pre- or intraoperatively advanced PDAC (*n* = 58 vs. *n* = 166, respectively; Table 1). The median CA19-9 and CEA serum levels differed significantly between the resected and (intra- and preoperative) advanced PDAC groups (CA19-9: 153.0, 243.5 476.3 kU/L and CEA: 3.2, 5.2 and 5.7 µg/L, respectively; Table 1). The median survival was 24 (17–32) months for the patients who underwent resection, which was significantly longer compared to both advanced PDAC groups with a median survivals of 7 (6–8) and 5 (4–6) months, respectively.

### 3.2. CA19-9 vs. CEA and Overall Survival

**Figure 1:**
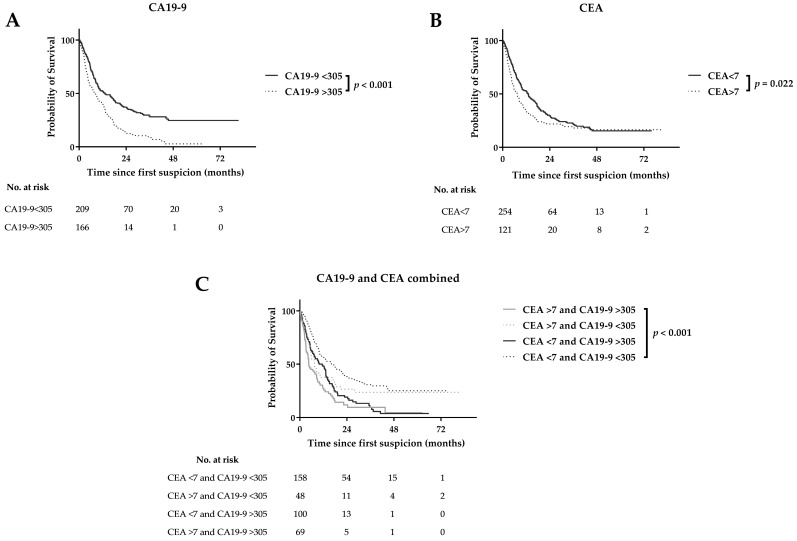
Survival curves for CA19-9 (**A**) CEA (**B**) and combined CA19-9 and CEA (**C**) serum levels.

In the entire cohort, both CA19-9 >27 kU/L and >305 kU/L were statistically significant associated with poor OS (median OS: 10 (8–11) versus 22 (6–37) months; 8 (6–10) vs. 13 (8–18) months, respectively; Figure 1A). CEA >7 µg/L was statistically significant associated with poor OS (7 (6–9) versus 13 (10–16) months), whereas a cutoff value of 3 µg/L showed similar median OS (Figure 1B). In the resection cohort, CA19-9>27 kU/L and CA19-9 >305 kU/L were statistically significant associated with poor OS (22 (17–27) versus 44 (35–53) months; 17 (14–20) versus 44 (28–60) months, respectively), whereas elevated CEA levels were not associated with poor OS (Figure S1). In the pre- and intraoperatively advanced PDAC cohorts, both elevated CA19-9 and CEA levels were not statistically significant associated with poor OS (Figure S1). Combining elevated CA19-9 (>305 kU/L) and CEA (>7 µg/L) levels result in a median OS of 5 (3–6) months for the entire cohort, which was statistically significant worse compared to solely elevated CEA (10 (5–15) months), solely elevated CA19-9 (7 (5–10) months) or low serum levels for both markers (16 (11–21) months; Figure 1C).

At multivariate analysis, ASA score III-IV (HR: 1.74 (1.15–2.66)), CA19-9>305 kU/L (HR: 1.72 (1.31–2.26)) and tumor size >20 mm (HR: 2.36 (1.49–3.73)) were prognostic factors for poor OS in the entire cohort (Table 2). Combined elevated CA19-9 and CEA levels at multivariate analysis, showed that both elevated CA19-9 and CEA (HR: 1.71 (1.21–2.42)) and solely elevated CA19-9 (HR: 2.17 (1.46–3.23)) serum levels were independently associated with poor OS in the entire cohort (Table S1). In the resection cohort, CA19-9 > 305 kU/L was an independent prognostic factor for poor OS (HR: 2.59 (1.52–4.42)). As univariate analysis showed similar OS for pre- and intraoperatively advanced PDAC patients, both cohorts were combined at multivariate analysis. In this advanced PDAC cohort, CA19-9 > 305 kU/L was not an independent prognostic factor for poor OS (HR: 0.89 (0.64–1.24); Table 2).

### 3.3. CA19-9 and CEA and Time to Recurrence after Resection 

**Figure 2:**
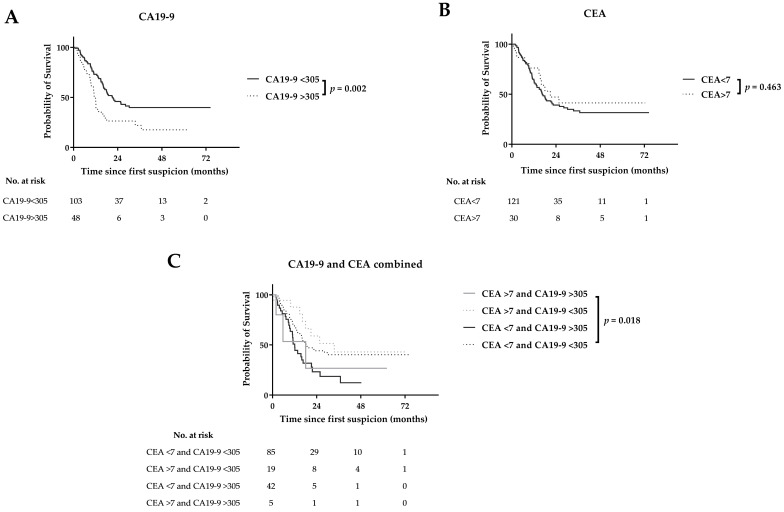
Recurrence patterns by CA19-9 (**A**), CEA (**B**), and combined CA19-9 and CEA (**C**) serum levels.

Median time to recurrence after resection was similar in patients with normal and elevated CEA (>3 µg/L; >7 µg/L) levels. CA19-9 >27 kU/L was not statistically significant associated with early recurrence (15 (11–18) versus 22 (12–32) months), whereas CA19-9 >305 kU/L was prognostic for early recurrence (11 (10–13) versus 21 (14–28) months), which was confirmed at multivariable analysis (Figure 2; Table 3). Combining elevated CA19-9 and CEA levels in multivariate analysis was not prognostic for early recurrence (Table S2). Furthermore, CA19-9 >305 kU/L was statistically significant associated with both early locoregional recurrence and distant metastases (Figure S2), although this could not be confirmed at multivariate analysis (Table 3).

### 3.4. Systematic Literature Review and Meta-Analysis

A total of 113 studies was identified and screened for eligibility (Figure S3). Eighteen studies, including ours, reported on the prognostic value determined at diagnosis of either CEA, CA19-9 serum levels or both on survival in PDAC patients (Table S3) [7,16,17,18,19,20,21,22,23,24,25,26,27,28,29]. Several cut-off values were used varying from 5–4000 kU/L for CA19-9 and 3–20 µg/L for CEA. Four studies did not perform multivariable analysis and were therefore not included in the meta-analysis [18,19,24,25]. The included studies demonstrated symmetric funnel plots (Figure S4). Pooled analysis showed a HR of 1.29 (1.17–1.42) and 1.51 (1.33–1.73) for elevated CA19-9 and CEA levels, respectively (Table 4). Pooled analysis of two studies, that combined elevated CA19-9 and CEA levels, showed a HR of 1.35 (1.33–1.37; Table S4). Subgroup analysis showed similar results in both advanced (*n* = 8 studies) and resected *n* = 10 studies) patients.

Four studies reported on the prognostic value for recurrence after pancreatic resections for PDAC patients of preoperative serum CA19-9 levels, whereas no studies evaluated the prognostic value of CEA, except ours (Table S5) [7,8,9,21]. In three studies, including ours, elevated CA19-9 levels (CA19-9 > 37 kU/L, CA19-9 > 100 kU/L and CA19-9 > 305 kU/L) were a prognostic factor for early recurrence (pooled HR: 2.41 (1.77–3.29)), whereas two studies did not perform a multivariable analysis (Table 4).

## 4. Discussion

This study aimed to determine the stage-specific prognostic value of CA19-9 and CEA serum levels at diagnosis on OS and time to recurrence in PDAC patients discussed at MDT meetings. In the current cohort, both elevated CA19-9 (>305 kU/L) and CEA (>7 µg/L) levels were significantly associated with a poor median OS. Multivariable analysis showed that CA19-9 is an independent prognostic factor (HR: 1.72) for poor OS, whereas CEA did not yield a significant association anymore. Moreover, elevated CA19-9 levels (>305 kU/L) were independently associated with early recurrence (HR: 1.74). Our systematic literature search revealed that eighteen studies reported on either the prognostic value of both CEA and CA19-9 for OS, time to recurrence or both in PDAC patients, although none of them included consecutive PDAC patients discussed at the MDT meetings. Generally, the findings of these studies were in concordance with our analyses, as the meta-analysis revealed a prognostic role of elevated CA19-9 (pooled HR: 1.29) and CEA levels (pooled HR: 1.51) on OS. However, no optimal cut-off values for CA19-9 and CEA could be determined, as the included studies used a variety of cut-off values.

One of the limitations of our cohort study is its retrospective design. Because of missing values CEA and CA19-9 were imputed several times based on relevant prognostic factors. Moreover, elevated CEA levels could also be elevated in case of nicotine abuses or presence of other cancers, such as colon cancer or rare adeno-squamous PDAC [30,31,32]. In patients missing the Lewis antigen, which is the case in 4–7% of the population, CA19-9 level will stay low, therefore both CA19-9 and CEA levels should be interpreted with caution [33,34,35]. Elevated bilirubin serum levels as result of biliary obstruction could also influence the serum CA19-9 levels, although in this study we corrected for this effect during the analyses [36]. Compared to the meta-analysis, elevated CEA levels were not associated with poor OS or early recurrence in our cohort. Publication bias and the use of different cut-off levels could be a possible explanation of the discrepancy between the outcomes of the studies included in the meta-analysis and our retrospective cohort, although the shape of the funnel plots, including only five studies (including ours), was symmetric.

Tailored treatment of the individual cancer patient is the future in surgical oncology. In the era of neoadjuvant treatment for pancreatic cancer, we do not know in advance which patient will benefit or not. It has been shown that a >30% decrease of CA19-9 levels is related with a response on neoadjuvant chemotherapy thereby improving the prediction rate for resectability of LAPC, illustrating that tumor biology, and not only tumor anatomy, plays an important role [37]. Furthermore, monitoring of disease recurrence after resection by testing biomarkers could be helpful in addition to imaging techniques such as CT, MRI or PET-CT [38,39]. CA 19-9 has been shown superior to CEA for monitoring of recurrence following radical resection of pancreatic cancer [40]. The results of this study could implicate that the MDT should consider neoadjuvant therapy in patients with elevated CA19-9 levels to achieve local downstaging in order to prevent patients from early recurrence and therefore unnecessary and high impact surgeries with long recovery times. Moreover, the results of the meta-analysis suggest that both elevated CA19-9 and CEA levels could indicate aggressive tumor growth in advanced stage PDAC, resulting in a poor OS.

## 5. Conclusions

In conclusion, elevated CA19-9 serum levels appear to be an independent prognostic factor for poor OS in PDAC patients, whereas the prognostic value of CEA is disputable. In patients who underwent resection, preoperative CA19-9 and not CEA levels are prognostic for early recurrence, which could be a plea to consider them as biologically (locally) advanced disease. Future trials should standardize and incorporate CA19-9 levels in their patient and treatment selection allocation.

## Figures and Tables

**Table 1:** Patient and tumor characteristics.

Variable	Resected PDAC (*n* = 151)	Intraoperative Advanced PDAC (*n* = 58)	Preoperative Advanced PDAC (*n* = 166)	*p*-Value
Age (y), mean (SD)	64.8 (9.8)	66.1 (10.1)	67.8 (9.6)	0.021
Sex, *n* (%)				0.206
Male	80 (53.0)	25 (43.1)	94 (56.6)	
Female	71 (47.0)	33 (56.9)	72 (43.4)	
ASA score, *n* (%)				0.360
I	21 (13.9)	7 (12.1)	34 (20.5)	
II	99 (65.6)	39 (67.2)	95 (57.2)	
III-IV	31 (20.5)	12 (20.7)	37 (22.3)	
Bilirubin (μmol/L), mean (SD)	114.9 (129.8)	89.1 (120.2)	85.7 (127.5)	0.143
Tumormarkers, median (IQR)				
CA19-9 (kU/L)	153.0 (30.5–520.8)	243.5 (66.8–678.3)	476.3 (107.9–2145.3)	<0.001
CEA (µg/L)	3.2 (2.0–4.8)	5.2 (3.3–16.3)	5.7 (2.6–14.6)	<0.001
Tumormarkers available, *n* (%)				
CA19-9	121 (80.1)	50 (86.2)	130 (78.3)	0.431
CEA	90 (59.6)	41 (70.7)	86 (51.8)	0.037
Tumor location, *n* (%)				0.044
Head	119 (78.8)	44 (75.9)	106 (63.9)	
Body	17 (11.3)	9 (15.5)	36 (21.7)	
Tail	15 (9.9)	5 (8.6)	24 (14.5)	
Preoperative TNM stage, *n* (%)				<0.001
Ia	23 (15.2)	4 (6.9)	0	
Ib	34 (22.5)	7 (12.1)	0	
IIa	75 (49.7)	33 (56.9)	7 (4.2)	
IIb	18 (11.9)	12 (20.7)	2 (1.2)	
III	1 (0.7)	2 (3.4)	65 (39.2)	
IV	0	0	92 (55.4)	
Tumor size (mm), mean (SD)	27.9 (12.8)	37.8 (17.3)	40.3 (17.5)	<0.001
Chemotherapy, *n* (%)				<0.001
No or unknown	36 (23.8)	41 (70.6)	108 (65.1)	
Yes	115 (76.2)	17 (29.4)	58 (34.9)	
Neoadjuvant	7	-	6	
Adjuvant	115	-	-	
Palliative	-	17	52	
Survival (months), median (95%CI)	24 (17–32)	7 (6–8)	5 (4–6)	<0.001

Abbreviations: PDAC: pancreatic ductal adenocarcinoma; CA19-9: carbohydrate antigen 19-9; CEA: carcinoembryonic antigen.

**Table 2:** Multivariable cox regression analysis for overall survival.

Parameter	Entire Cohort (*n* = 375)			Resected Cohort (*n* = 151)			Advanced PDAC Cohort (*n* = 224)		
	HR	95% CI	*p*-Value	HR	95% CI	*p*-Value	HR	95% CI	*p*-Value
Tumormarkers									
CA19-9 > 305 kU/L	1.72	1.31–2.26	<0.001	2.59	1.52–4.42	<0.001	0.89	0.64–1.24	0.494
CEA > 7 µg/L	1.26	0.89–1.77	0.191	0.66	0.29–1.46	0.302	1.11	0.79–1.49	0.568
Age (yr)									
>65 yr	1.28	0.98–1.67	0.067	1.03	0.66–1.63	0.891	1.01	0.72–1.42	0.964
Sex, *n* (%)									
Female	0.84	0.65–1.08	0.170	1.01	0.64–1.60	0.960	0.65	0.47–0.88	0.006
ASA score, *n* (%)									
I	1.00	1.00–1.00	1.000	1.00	1.00–1.00	1.000	1.00	1.00–1.00	1.000
II	1.13	0.78–1.63	0.531	1.50	0.68–3.29	0.315	1.64	1.05–2.56	0.028
III-IV	1.74	1.15–2.66	0.010	3.44	1.45–8.13	0.005	2.28	1.34–3.86	0.002
Bilirubin									
>17 μmol/L	0.78	0.61–1.01	0.062	0.81	0.50–1.31	0.383	1.09	0.79–1.49	0.612
Tumor size									
>20 mm	2.36	1.49–3.73	<0.001	1.57	0.81–3.06	0.184	2.74	1.37–5.49	0.004

Abbreviations: CA19-9: carbohydrate antigen 19-9; CEA: carcinoembryonic antigen.

**Table 3:** Multivariable cox regression analysis for recurrence in resected PDAC cohort.

Parameter	General Recurrence (*n* = 84) *			Locoregional Recurrence (*n* = 55) *			Distant Recurrence (*n* = 61) *		
	HR	95% CI	*p*-Value	HR	95% CI	*p*-Value	HR	95% CI	*p*-Value
Tumormarkers									
CA19-9 > 305 kU/L	1.72	1.03–2.85	0.038	1.81	0.96–3.42	0.067	1.75	0.95–3.20	0.072
CEA > 7 µg/L	0.70	0.30–1.63	0.413	0.77	0.25–2.36	0.650	0.73	0.27–1.96	0.536
Tumor size									
>20 mm	1.95	1.02–3.72	0.043	1.76	0.83–3.77	0.144	1.76	0.83–3.71	0.140
Perineural invasion									
Present	1.24	0.77–2.02	0.376	1.12	0.62–2.02	0.715	1.38	0.78–2.46	0.273
Margin status									
R1	1.46	0.93–2.29	0.099	1.38	0.79–2.41	0.253	1.59	0.95–2.67	0.079
Differentiation grade									
Well	1.00	1.00–1.00	1.000	1.00	1.00–1.00	1.000	1.00	1.00–1.00	1.000
Moderate	1.88	0.91–3.86	0.087	1.61	0.74–3.52	0.233	2.04	0.82–5.07	0.124
Poorly	1.80	0.88–3.71	0.111	1.05	0.46–2.41	0.904	2.17	0.88–5.34	0.093
Undifferentiated or unknown	1.64	0.50–5.35	0.415	0.66	0.14–3.16	0.600	2.62	0.72–9.57	0.144

Abbreviations: PDAC: pancreatic ductal adenocarcinoma; HR: Hazard Ratio; CA19-9: carbohydrate antigen 19-9; CEA: carcinoembryonic antigen. * Some patients had both locoregional recurrence and distant metastases.

**Table 4:** Forest plots of prognostic value of (**A**) CA19-9 and (**B**) CEA on overall survival in PDAC patients, including subgroup analysis. The prognostic value of CA19-9 on recurrence in resected PDAC patients was also shown (**C**).

Reference	Patients (*n*)	Hazard Ratio (95% CI)	Hazard Ratio
**A** Prognostic Value CA19–9 on Survival			
Advanced disease cohort			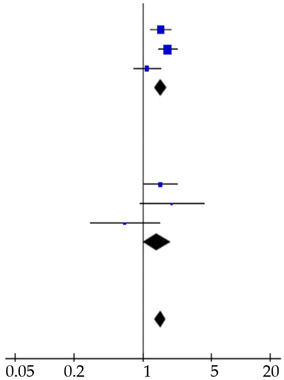
Reitz et al., 2015 [26]	288	1.50 (1.17–1.91)	
Imaoka et al., 2016 [20]	433	1.09 (0.81–1.47)	
Song et al., 2018 [28]	59	2.22 (1.24–3.98)	
Tingle et al., 2018 [29]	115	2.70 (1.10–6.63)	
This study	224	0.89 (0.64–1.24)	
Subtotal	1119	1.28 (1.10–1.50)	
Heterogeneity: χ^2^ = 13.43, d.f. = 4, *p* = 0.009; I^2^ = 70%. Test for overall effect: Z = 3.13, *p* = 0.002			
Resected cohort			
Smith et al., 2008 [27]	109	1.17 (1.02–1.35)	
Lee et al., 2013 [23]	187	0.88 (0.57–1.35)	
Dong et al., 2014 [17]	139	1.96 (1.24–3.10)	
Reitz et al., 2015 [26]	105	2.50 (1.51–4.15)	
Asaoka et al., 2016 [7]	46	3.71 (1.23–11.16)	
Kondo et al., 2017 [22]	198	0.84 (0.34–2.09)	
This study	151	2.59 (1.52–4.41)	
Subtotal	935	1.30 (1.15–1.46)	
Heterogeneity: χ^2^ = 25.62, d.f. = 6, *p* < 0.001; I^2^ = 77%. Test for overall effect: Z = 4.27, *p* < 0.001			
Total	2054	1.29 (1.17–1.42)	
Heterogeneity: χ^2^ = 39.06, d.f. = 11, *p* < 0.001; I^2^ = 72% Test for overall effect: Z = 5.30, *p* < 0.001 Test for subgroup differences: χ^2^ = 0.01, d.f. = 1, *p* = 0.91; I^2^ = 0%			
**B** Prognostic value CEA on survival			
Advanced disease cohort			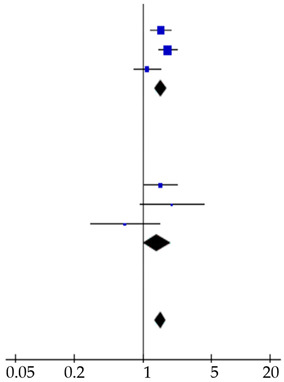
Reitz et al., 2015 [26]	288	1.54 (1.20–1.97)	
Imaoka et al., 2016 [20]	433	1.81 (1.45–2.26)	
This study	224	1.11 (0.81–1.52)	
Subtotal	945	1.54 (1.33–1.78)	
Heterogeneity: χ^2^ = 6.13, d.f. = 2, *p* = 0.05; I^2^ = 67%. Test for overall effect: Z = 5.78, *p* < 0.001			
Resected cohort			
Lee et al., 2013 [23]	187	1.52 (1.03–2.24)	
Reitz et al., 2015 [26]	105	1.99 (0.94–4.22)	
This study	151	0.66 (0.29–1.48)	
Subtotal	443	1.40 (1.02–1.92)	
Heterogeneity: χ^2^ = 25.62, d.f. = 6, *p* < 0.001; I^2^ = 77%. Test for overall effect: Z = 4.27, *p* < 0.001			
Total	1388	1.51 (1.33–1.73)	
Heterogeneity: χ^2^ = 10.74, d.f. = 5, *p* = 0.06; I^2^ = 53%. Test for overall effect: Z = 6.13, *p* < 0.001 Test for subgroup differences: χ^2^ = 0.27, d.f. = 1, *p* = 0.60; I^2^ = 0%			
**C** Prognostic value CA19–9 on recurrence			
Waraya et al., 2009 [9]	117	2.16 (1.39–3.36)	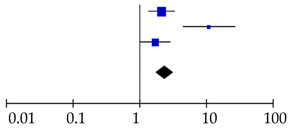
Sugiura et al., 2012 [8]	154	11.19 (4.55–27.5)	
This study	151	1.74 (1.06–2.86)	
Total	422	2.41 (1.77–3.29)	
Heterogeneity: χ^2^ = 13.09, d.f. = 2, *p* = 0.001; I^2^ = 85%. Test for overall effect: Z = 5.58, *p* < 0.001			

Abbreviations: CA19-9: carbohydrate antigen 19-9; CEA: carcinoembryonic antigen; d.f.: degrees of freedom.

## Supplementary Materials

The following are available online at https://www.mdpi.com/2072-6694/12/10/2970/s1, Figure S1: Survival curves for CA19-9 and CEA serum levels stratified by resected PDAC (A–C), intraoperative advanced PDAC (D–F) and preoperative advanced PDAC (G–I) patients, Figure S2. Recurrence patterns (locoregional (A–C) versus metastatic recurrence (D–F)) by CA19-9 and CEA serum levels, Figure S3: Flowchart of study selection, Figure S4: Funnel plots of included studies in meta-analysis, Table S1: Multivariable cox regression analysis for overall survival including combined CA19-9 and CEA levels, Table S2: Multivariable cox regression analysis for recurrence in resected PDAC cohort including combined CA19-9 and CEA levels, Table S3: Studies that report on prognostic value of serum CA19-9 and CEA on overall survival in PDAC patients, Table S4: Forest plot of prognostic value of CA19-9 and CEA combined on overall survival in PDAC patients, Table S5. Studies that report on prognostic value of preoperative serum CA19-9 and CEA for early recurrence in resected PDAC patients.
