# Supplementary material for: Stage-Specific Value of Carbohydrate Antigen 19-9 and Carcinoembryonic Antigen Serum Levels on Survival and Recurrence in Pancreatic Cancer: A Single Center Study and Meta-Analysis
Source: Cancers (Basel). 2020 Oct 14;12(10):2970. doi: 10.3390/cancers12102970 (PMC7602123; doi:10.3390/cancers12102970)
Supplement: Supplementary file 1 [file cancers-12-02970-s001.pdf]

cancers

MDPI

Article

Stage-Specific Value of Carbohydrate Antigen 19-9 and Carcinoembryonic Antigen Serum Levels on Survival and Recurrence in Pancreatic Cancer: A Single Center Study and Meta-Analysis

Labrinus van Manen, Jesse V. Groen, Hein Putter, Martin Pichler, Alexander L. Vahrmeijer, Bert A. Bonsing, and J. Sven D. Mieog

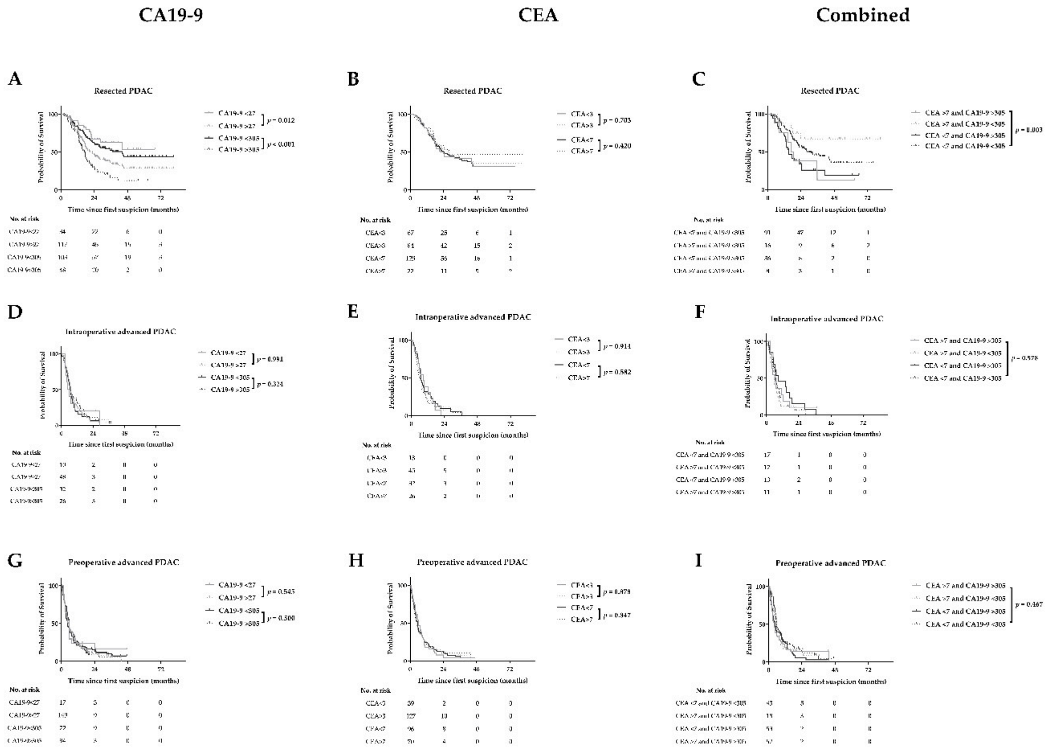

Figure S1. Survival curves for CA19-9 and CEA serum levels stratified by resected PDAC (**A–C**), intraoperative advanced PDAC (**D–F**) and preoperative advanced PDAC (**G–I**) patients.

Table S1. Multivariable cox regression analysis for overall survival including combined CA19-9 and CEA levels.

**Parameter**	**Entire Cohort (*n* = 375)**			**Resected Cohort (*n* = 151)**			**Advanced PDAC Cohort (*n* = 224)**		
	**HR**	**95% CI**	***p*-value**	**HR**	**95% CI**	***p*-value**	**HR**	**95% CI**	***p*-value**
Tumormarkers									
CA19-9 < 305 kU/L and CEA < 7 µg/L	1.00	1.00–1.00	1.000	1.00	1.00-1.00	1.000	1.00	1.00–1.00	1.000
CA19-9 < 305 kU/L and CEA >7 µg/L	1.24	0.72–2.12	0.439	0.40	0.11–1.38	0.145	1.30	0.76–2.21	0.337
CA19-9 > 305 kU/L and CEA < 7 µg/L	1.71	1.21–2.42	0.003	2.23	1.24–4.01	0.007	1.00	0.65–1.54	0.992
CA19-9 >305 kU/L and CEA > 7 µg/L	2.17	1.46–3.23	<0.001	2.74	0.86–8.70	0.088	0.99	0.62–1.58	0.967
Age (yr)									
>65 yr	1.28	0.98–1.67	0.070	1.03	0.65–1.64	0.889	1.02	0.72–1.44	0.917
Sex, *n* (%)									
Female	0.84	0.65–1.08	0.167	0.98	0.62–1.57	0.943	0.65	0.47–0.88	0.006
ASA score, *n* (%)									
I	1.00	1.00–1.00	1.000	1.00	1.00–1.00	1.000	1.00	1.00–1.00	1.000
II	1.13	0.78–1.65	0.526	1.69	0.73–3.91	0.224	1.61	1.03–2.52	0.038
III-IV	1.75	1.15–2.67	0.010	3.85	1.53–9.69	0.004	2.23	1.31–3.79	0.003
Bilirubin									
>17 µmol/L	0.78	0.61–1.01	0.063	0.79	0.48–1.30	0.351	1.09	0.80–1.51	0.581
Tumor size									
>20 mm	2.36	1.48–3.75	<0.001	1.60	0.82–3.13	0.170	2.73	1.36–5.50	0.005

Abbreviations: CA19-9: carbohydrate antigen 19-9; CEA: carcinoembryonic antigen.

Table S2. Multivariable cox regression analysis for recurrence in resected PDAC cohort including combined CA19-9 and CEA levels.

Parameter	General Recurrence (*n* = 84) *			Locoregional Recurrence (*n* = 55) *			Distant Recurrence (*n* = 61) *		
	HR	95% CI	*p*-value	HR	95% CI	*p*-value	HR	95% CI	*p*-value
Tumormarkers									
CA19-9 <305 kU/L and CEA<7 μg/L	1.00	1.00–1.00	1.000	1.00	1.00–1.00	1.000	1.00	1.00–1.00	1.000
CA19-9 <305 kU/L and CEA >7 μg/L	0.62	0.23–1.70	0.354	0.66	0.17–2.60	0.551	0.65	0.21–2.00	0.449
CA19-9 > 305 kU/L and CEA < 7 μg/L	1.63	0.93–2.86	0.091	1.70	0.83–3.48	0.149	1.65	0.85–3.21	0.140
CA19-9 >305 kU/L and CEA >7 μg/L	1.41	0.40–5.00	0.591	1.44	0.51–4.02	0.491	1.49	0.31–7.09	0.614
Tumor size									
>20 mm	1.97	1.03–3.76	0.041	1.79	0.83–3.83	0.136	1.77	0.84–3.74	0.135
Perineural invasion									
Present	1.26	0.77–2.07	0.353	1.13	0.62–2.06	0.692	1.41	0.78–2.53	0.257
Margin status									
R1	1.48	0.94–2.32	0.092	1.40	0.80–2.46	0.242	1.61	0.95–2.71	0.075
Differentiation grade									
Well	1.00	1.00–1.00	1.000	1.00	1.00–1.00	1.000	1.00	1.00–1.00	1.000
Moderate	1.88	0.91–3.86	0.088	1.61	0.73–3.53	0.234	2.04	0.82–5.08	0.125
Poorly	1.66	0.51–5.41	0.109	1.06	0.46–2.44	0.895	2.18	0.88–5.39	0.091
Undifferentiated or unknown	1.66	0.51–5.41	0.405	0.65	0.14–3.18	0.599	2.67	0.73–9.76	0.138

Abbreviations: PDAC: pancreatic ductal adenocarcinoma; HR: Hazard Ratio; CA19-9: carbohydrate antigen 19-9; CEA: carcinoembryonic antigen. * Some patients had both locoregional recurrence and distant metastases.

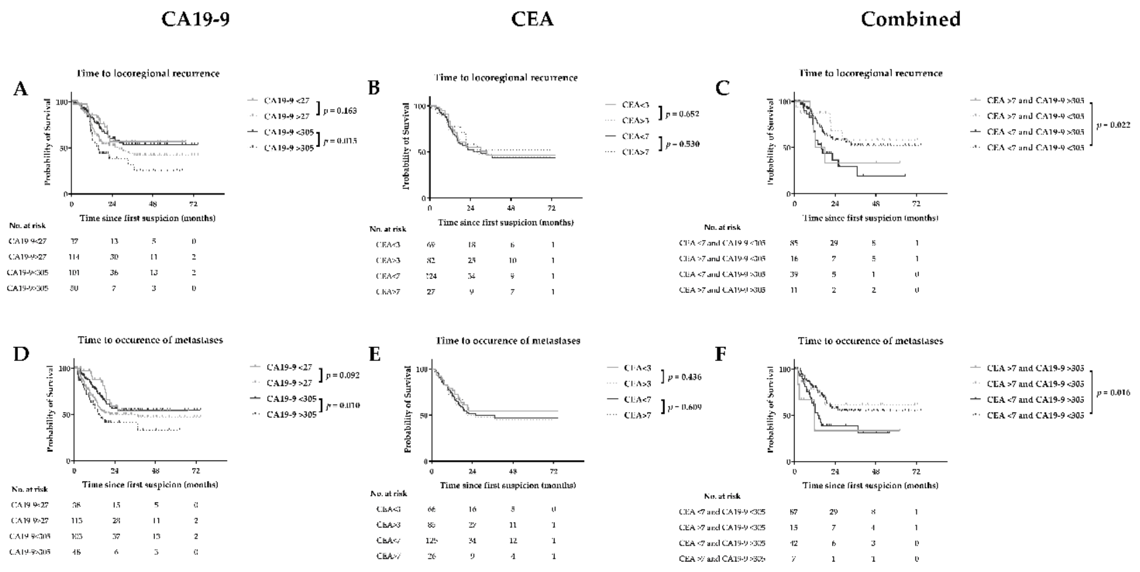

Figure S2. Recurrence patterns (locoregional (**A–C**) vs metastatic recurrence (**D–F**)) by CA19-9 and CEA serum levels.

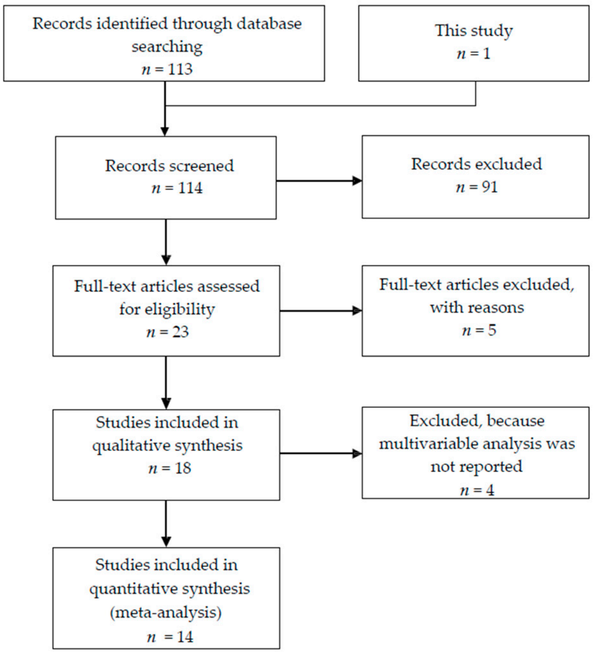

Figure S3. Flowchart of study selection.

Table S3. Studies that report on prognostic value of serum CA19-9 and CEA on overall survival in PDAC patients.

Study	Country	Study Population (No. of Patients)	Cut-off Level (CEA: µg/L; CA19-9: kU/L)	Fraction above Cut-off (%)	Median OS (95% CI) below Cut-off (Months)	Median OS (95% CI) above Cut-off (Months)	Performance of Multivariable analysis
CA19-9							
Ni et al., 2005 [1]	China	Resected and advanced PDAC (*n* = 105)	1,000	37.8	9	6	No
Ferrone et al., 2006 [2]*	United States	Resected PDAC (*n* = 111)	37<br>200<br>1000<br>2000	NR	28<br>28<br>28<br>22	19<br>14<br>12<br>13	No
Smith et al., 2008 [3]	United Kingdom	Resected PDAC (*n* = 109)	150	58.7	22.1	10.4	Yes
Martin et al., 2012 [4] *	United States	Resected (*n* = 30) and advanced (*n* = 93) PDAC	37	Resected: 50<br>Advanced: 72	Resected: 36<br>Advanced: 32.3	Resected: 20<br>Advanced: 11.3	No
Hartwig et al., 2013 [5] *	Germany	Resected PDAC (*n* = 1543)	<5<br>5–37<br>37–100<br>100–250<br>250–500<br>500–1,000<br>1000–2,000<br>2000–4,000<br>≥4000	6.4<br>18.2<br>14.0<br>16.0<br>13.2<br>11.9<br>8.2<br>6.0<br>6.1	-	26.8<br>28.5<br>26.9<br>22.5<br>20.1<br>15.4<br>12<br>12.3<br>14.4	No
Lee et al., 2013 [6]	Korea	Resected PDAC (*n* = 187)	376	75.4	12.4	13.5	Yes
Dong et al., 2014 [7]	China	Resected PDAC (*n* = 139)	339	43.3	24.9	11.9	Yes
Reitz et al., 2015 [8]	Austria	Resected and advanced PDAC (*n* = 393)	931	47.1	10	5	Yes
Asaoka et al., 2016 [9]	Japan	Resected PDAC (*n* = 46)	230	30.4	NR	15.8	Yes
Imaoka et al., 2016 [10]	Japan	Resected PDAC (*n* = 119)	37	68.0	55.9	44.9	Yes
Imaoka et al., 2016 [11]	Japan	Metastatic PDAC (*n* = 433)	37	84.8	8.9	7.8	Yes

Kondo et al., 2017 [12]	Japan	Resected PDAC (*n* = 198)	300	32.2	18.8	46.7	Yes
Tingle et al., 2018 [13]	United Kingdom	Advanced PDAC (*n* = 115)	770	NR	15.1	7.7	Yes
Song et al., 2018 [14]	China	Metastatic PDAC (*n* = 59)	626	57.6	NR	NR	Yes
This study	The Netherlands	Resected and advanced PDAC (*n* = 375)	305	44.3	13	8	Yes
CEA							
Ni et al., 2005 [1]	China	Resected and advanced PDAC (*n* = 105)	5	47.6	11	6	No
Lee et al., 2013 [6]	Korea	Resected PDAC (*n* = 187)	5	39.0	16.1	10.2	Yes
Reitz et al., 2015 [8]	Austria	Resected and advanced PDAC (*n* = 393)	6.9	33.6	10	4	Yes
Asaoka et al., 2016 [9]	Japan	Resected PDAC (*n* = 46)	4	23.9	NR	NR	Yes
Imaoka et al., 2016 [11]	Japan	Metastatic PDAC (*n* = 433)	5<br>20	57.3<br>30.3	10.3<br>NR	6.8<br>6.1	Yes
This study	The Netherlands	Resected and advanced PDAC (*n* = 375)	7	32.3	13	7	Yes
CA19-9 and CEA combined							
Distler et al., 2013 [15]	Germany	Resected PDAC (*n* = 259)	75 (CA19-9)<br>3 (CEA)	35.1	33.3	23.9	Yes
This study	The Netherlands	Resected and advanced PDAC (*n* = 375)	305 (CA19-9)<br>7 (CEA)	18.4	7	5	Yes

CA 19-9: cancer antigen 19-9, CEA: carcinoembryonic antigen, OS: overall survival, PDAC: pancreatic ductal adenocarcinoma, HR: Hazard Ratio, NR: not reported. * Multiple cut-off values have been used.

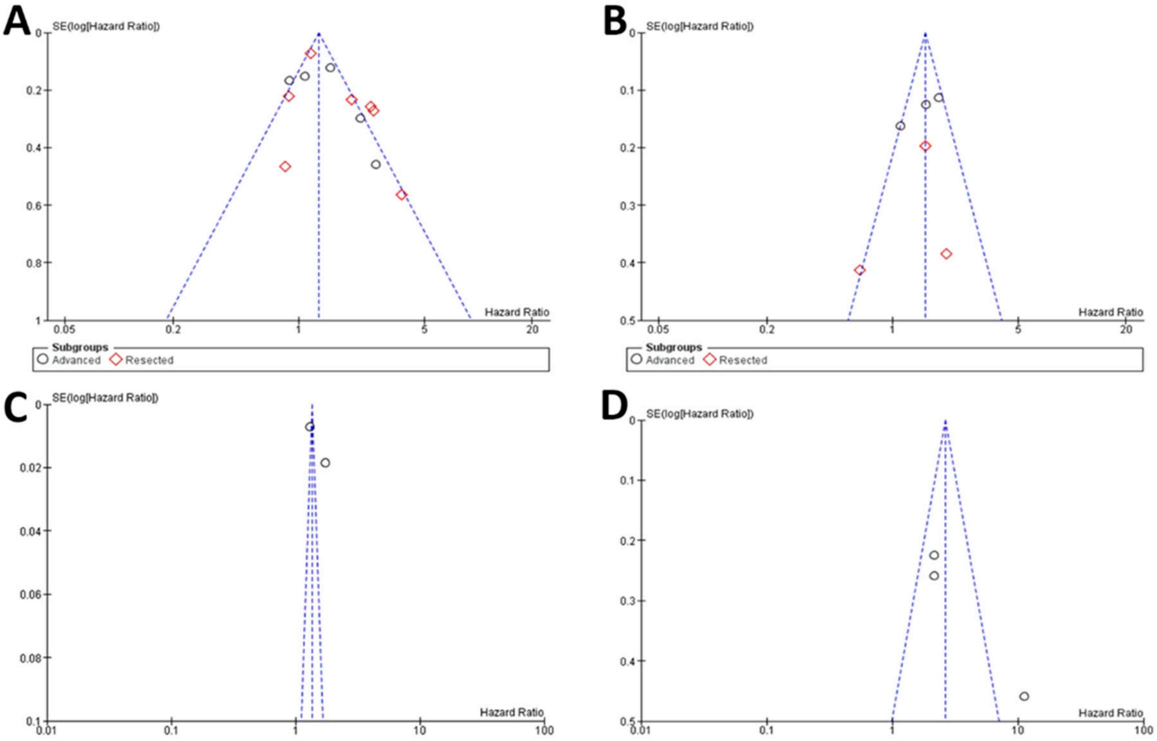

Figure S4. Funnel plots of included studies in meta-analysis, determining the prognostic value of CA19-9 on overall survival (**A**) CEA on overall survival (**B**) both CA19-9 and CEA on overall survival (**C**) and CA19-9 on recurrence (**D**) in PDAC patients.

Table S4. Forest plot of prognostic value of CA19-9 and CEA combined on overall survival in PDAC patients.

Reference	Patients (*n*)	Hazard Ratio (95% CI)	Hazard Ratio
Distler et al., 2013 [15]	259	1.30 (1.13–1.50)	
This study	375	1.73 (1.20–2.48)	
Total	634	1.35 (1.33–1.37)	
Heterogeneity: $\chi^2 = 206.50$, d.f. = 1, $p < 0.001$; $I^2 = 100\%$			
Test for overall effect: $Z = 44.58$, $p < 0.001$			

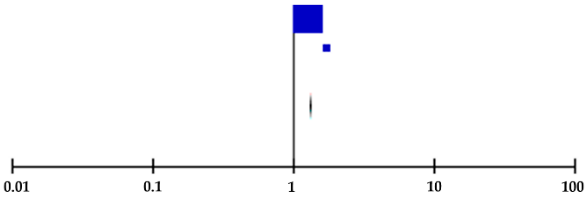

CA 19-9: cancer antigen 19-9; CEA: carcinoembryonic antigen; d.f.: degrees of freedom.

Table S5. Studies that report on prognostic value of preoperative serum CA19-9 and CEA for early recurrence in resected PDAC patients.

Study	Country	Marker	Study Population (No. of Patients)	Cut-off Level (CEA: µg/L; CA19-9: kU/L)	Fraction above Cut-off (%)	Median time-to-Recurrence (95% CI) below Cut-off (Months)	Median time-to-Recurrence (95% CI) above Cut-off (months)	Performance of Multivariable Analysis
Waraya et al., 2009 [16]	Japan	CA19-9	Resected PDAC (*n* = 117)	37	56.4	30.6	12.7	Yes
Sugiura et al., 2012 [17]	Japan	CA19-9	Resected PDAC (*n* = 154)	100	47.4	22	6	Yes
Asaoka et al., 2016 [9] *	Japan	CA19-9	Resected PDAC (*n* = 46)	230	30.4	12.7	5.8	No
Imaoka et al., 2016 [10] *	Japan	CA19-9	Resected PDAC (*n* = 119)	37	68.0	24.8	17.9	No
This study	The Netherlands	CA19-9	Resected PDAC (*n* = 151)	27<br>305	76.8<br>31.8	22<br>21	15<br>11	Yes
		CEA	Resected PDAC (*n* = 151)	3<br>7	53.0<br>19.9	18<br>21	15<br>16	No

CA 19-9: cancer antigen 19-9, CEA: carcinoembryonic antigen, PDAC: pancreatic ductal adenocarcinoma.

References

1. Ni, X.G.; Bai, X.F.; Mao, Y.L.; Shao, Y.F.; Wu, J.X.; Shan, Y.; Wang, C.F.; Wang, J.; Tian, Y.T.; Liu, Q.; et al. The clinical value of serum CEA, CA19-9, and CA242 in the diagnosis and prognosis of pancreatic cancer. *Eur. J. Surg. Oncol. J. Eur. Soc. Surg. Oncol. Br. Assoc. Surg. Oncol.* **2005**, *31*, 164–169, doi:10.1016/j.ejso.2004.09.007.
2. Ferrone, C.R.; Finkelstein, D.M.; Thayer, S.P.; Muzikansky, A.; Fernandez-delCastillo, C.; Warshaw, A.L. Perioperative CA19-9 levels can predict stage and survival in patients with resectable pancreatic adenocarcinoma. *J. Clin. Oncol. Off. J. Am. Soc. Clin. Oncol.* **2006**, *24*, 2897–2902, doi:10.1200/jco.2005.05.3934.
3. Smith, R.A.; Bosonnet, L.; Ghaneh, P.; Raraty, M.; Sutton, R.; Campbell, F.; Neoptolemos, J.P. Preoperative CA19-9 levels and lymph node ratio are independent predictors of survival in patients with resected pancreatic ductal adenocarcinoma. *Dig. Surg.* **2008**, *25*, 226–232, doi:10.1159/000140961.

4. Martin, L.K.; Wei, L.; Trolli, E.; Bekaii-Saab, T. Elevated baseline CA19-9 levels correlate with adverse prognosis in patients with early- or advanced-stage pancreas cancer. *Med. Oncol.* **2012**, *29*, 3101–3107, doi:10.1007/s12032-012-0278-9.
5. Hartwig, W.; Strobel, O.; Hinz, U.; Fritz, S.; Hackert, T.; Roth, C.; Buchler, M.W.; Werner, J. CA19-9 in potentially resectable pancreatic cancer: Perspective to adjust surgical and perioperative therapy. *Ann. Surg. Oncol.* **2013**, *20*, 2188–2196, doi:10.1245/s10434-012-2809-1.
6. Lee, K.J.; Yi, S.W.; Chung, M.J.; Park, S.W.; Song, S.Y.; Chung, J.B.; Park, J.Y. Serum CA 19-9 and CEA levels as a prognostic factor in pancreatic adenocarcinoma. *Yonsei Med. J.* **2013**, *54*, 643–649, doi:10.3349/ymj.2013.54.3.643.
7. Dong, Q.; Yang, X.H.; Zhang, Y.; Jing, W.; Zheng, L.Q.; Liu, Y.P.; Qu, X.J. Elevated serum CA19-9 level is a promising predictor for poor prognosis in patients with resectable pancreatic ductal adenocarcinoma: A pilot study. *World J. Surg. Oncol.* **2014**, *12*, 171, doi:10.1186/1477-7819-12-171.
8. Reitz, D.; Gerger, A.; Seidel, J.; Kornprat, P.; Samonigg, H.; Stotz, M.; Szkandera, J.; Pichler, M. Combination of tumour markers CEA and CA19-9 improves the prognostic prediction in patients with pancreatic cancer. *J. Clin. Pathol.* **2015**, *68*, 427–433, doi:10.1136/jclinpath-2014-202451.
9. Asaoka, T.; Miyamoto, A.; Maeda, S.; Tsujie, M.; Hama, N.; Yamamoto, K.; Miyake, M.; Haraguchi, N.; Nishikawa, K.; Hirao, M.; et al. Prognostic impact of preoperative NLR and CA19-9 in pancreatic cancer. *Pancreatol. Off. J. Int. Assoc. Pancreatol.* **2016**, *16*, 434–440, doi:10.1016/j.pan.2015.10.006.
10. Imaoka, H.; Shimizu, Y.; Senda, Y.; Natsume, S.; Mizuno, N.; Hara, K.; Hijioka, S.; Hieda, N.; Tajika, M.; Tanaka, T.; et al. Post-adjuvant chemotherapy CA19-9 levels predict prognosis in patients with pancreatic ductal adenocarcinoma: A retrospective cohort study. *Pancreatol. Off. J. Int. Assoc. Pancreatol. (Iap) ... [Et Al.]* **2016**, *16*, 658–664, doi:10.1016/j.pan.2016.04.007.
11. Imaoka, H.; Mizuno, N.; Hara, K.; Hijioka, S.; Tajika, M.; Tanaka, T.; Ishihara, M.; Hirayama, Y.; Hieda, N.; Yoshida, T.; et al. Prognostic impact of carcinoembryonic antigen (CEA) on patients with metastatic pancreatic cancer: A retrospective cohort study. *Pancreatol. Off. J. Int. Assoc. Pancreatol. (Iap) ... [Et Al.]* **2016**, *16*, 859–864, doi:10.1016/j.pan.2016.05.007.
12. Kondo, N.; Murakami, Y.; Uemura, K.; Nakagawa, N.; Takahashi, S.; Ohge, H.; Sueda, T. Comparison of the prognostic impact of pre- and post-operative CA19-9, SPan-1, and DUPAN-II levels in patients with pancreatic carcinoma. *Pancreatol. Off. J. Int. Assoc. Pancreatol.* **2017**, *17*, 95–102, doi:10.1016/j.pan.2016.10.004.
13. Tingle, S.J.; Severs, G.R.; Goodfellow, M.; Moir, J.A.; White, S.A. NARCA: A novel prognostic scoring system using neutrophil-albumin ratio and Ca19-9 to predict overall survival in palliative pancreatic cancer. *J. Surg. Oncol.* **2018**, *118*, 680–686, doi:10.1002/jso.25209.
14. Song, J.Y.; Chen, M.Q.; Guo, J.H.; Lian, S.F.; Xu, B.H. Combined pretreatment serum CA19-9 and neutrophil-to-lymphocyte ratio as a potential prognostic factor in metastatic pancreatic cancer patients. *Medicine* **2018**, *97*, e9707, doi:10.1097/md.0000000000009707.
15. Distler, M.; Pilarsky, E.; Kersting, S.; Grutzmann, R. Preoperative CEA and CA 19-9 are prognostic markers for survival after curative resection for ductal adenocarcinoma of the pancreas-A retrospective tumor marker prognostic study. *Int. J. Surg.* **2013**, *11*, 1067–1072, doi:10.1016/j.ijsu.2013.10.005.
16. Waraya, M.; Yamashita, K.; Katagiri, H.; Ishii, K.; Takahashi, Y.; Furuta, K.; Watanabe, M. Preoperative serum CA19-9 and dissected peripancreatic tissue margin as determiners of long-term survival in pancreatic cancer. *Ann. Surg. Oncol.* **2009**, *16*, 1231–1240, doi:10.1245/s10434-009-0415-7.
17. Sugiura, T.; Uesaka, K.; Kanemoto, H.; Mizuno, T.; Sasaki, K.; Furukawa, H.; Matsunaga, K.; Maeda, A. Serum CA19-9 is a significant predictor among preoperative parameters for early recurrence after resection of pancreatic adenocarcinoma. *J. Gastrointest. Surg. Off. J. Soc. Surg. Aliment. Tract* **2012**, *16*, 977–985, doi:10.1007/s11605-012-1859-9.

© 2020 by the authors. Licensee MDPI, Basel, Switzerland. This article is an open access article distributed under the terms and conditions of the Creative Commons Attribution (CC BY) license (http://creativecommons.org/licenses/by/4.0/).
